# A Novel M2e Based Flu Vaccine Formulation for Dogs

**DOI:** 10.1371/journal.pone.0077084

**Published:** 2013-10-02

**Authors:** Denis Leclerc, Marie Rivest, Cindy Babin, Constantino López-Macias, Pierre Savard

**Affiliations:** 1 Microbiology, Infectiology and Immunology (Infectious Disease Research Centre), Laval University, Quebec City, P. Quebec, Canada; 2 Medical Research Unit on Immunochemistry, Specialties Hospital, National Medical Centre “Siglo XXI”, Mexican Social Security Institute (IMSS), Mexico City, Mexico; 3 Neurosciences, Laval University, Quebec City, P. Quebec, Canada; The University of Adelaide, Australia

## Abstract

**Background:**

The USA 2004 influenza virus outbreak H3N8 in dogs heralded the emergence of a new disease in this species. A new inactivated H3N8 vaccine was developed to control the spread of the disease but, as in humans and swine, it is anticipated that the virus will mutate shift and drift in the dog population. Therefore, there is a need for a vaccine that can trigger a broad protection to prevent the spread of the virus and the emergence of new strains.

**Methodology and Principal Findings:**

The universal M2e peptide is identical in almost all the H3N8 influenza strains sequenced to date and known to infect dogs. This epitope is therefore a good choice for development of a vaccine to provide broad protection. Malva mosaic virus (MaMV) nanoparticles were chosen as a vaccine platform to improve the stability of the M2e peptide and increase its immunogenicity in animals. The addition of an adjuvant (OmpC) purified from *Salmonella typhi* membrane in the vaccine formulation increased the immune response directed to the M2e peptide significantly and enlarged the protection to include the heterosubtypic strain of influenza in a mouse model. An optimal vaccine formulation was also shown to be immunogenic in dogs.

**Conclusions and Significance:**

The MaMV vaccine platform triggered an improved immune response directed towards the universal M2e peptide. The adjuvant OmpC increased the immune response to the M2e peptide and protection to a heterosubtypic influenza strain that harbors a different M2e peptide in a mouse model. Antibodies generated by the vaccine formulation showed cross-reactivity with M2e peptides derived from influenza strains H9N2, H5N1 and H1N1. The vaccine formulation shows a potential for commercialization of a new M2e based vaccine in dogs.

## Introduction

Infection of dogs with influenza virus was first reported in North America in 2004 in 22 racing greyhounds at a Florida racetrack. A detailed study of this event revealed that a H3N8 virus of equine origin was responsible of this incident. Virological, serological and molecular evidence supports this interspecies transfer between these two unrelated mammals [1], which is considered a very rare event. The vaccine industry rapidly offered a canine influenza vaccine (CIV) comprised of split influenza H3N8 strain adjuvanted with alum [2] to control spread of the disease. The virus has now been found in dogs in at least 30 states of the United States, which suggests that the virus has now adapted to dogs permanently and that new cases are not just originating from occasional cross-over’s from horses to dogs [3]. Thus, in addition to migrating birds, swine, horses and even cats, dogs are a new reservoir of influenza virus from which a new strain could emerge and infect humans.

Although canine influenza virus has emerged only recently, it is likely that the virus will adapt rapidly, mutate, drift and shift with other influenza strains, leading to the emergence of new influenza strains that will not be neutralized by antibodies generated by animals vaccinated with the current CIV [4]. There is therefore a medical need to develop a broad-spectrum influenza vaccine that will insure better control of virus spread and the risk of shedding of new emerging influenza diseases to other dogs and human.

Ideally, to be effective against a broad spectrum of strains of influenza A, an efficient CIV should trigger an immune response to highly conserved epitopes found in most influenza isolates. The sequence of the M2e peptide located at the N-terminus of the M2 protein—a membrane protein found associated with the virus particles and at the surface of influenza-infected cells [5–7] of influenza A has remained highly conserved across all influenza A isolates, thus making it an attractive epitope for the development of a vaccine with a broad spectrum of protection to disease.

The use of the M2e peptide alone as a vaccine antigen remains a major challenge because peptides are unstable and not immunogenic [8]. However, fusion of the M2e peptide to different vaccine platforms has allowed stabilization of the peptide and improvement of the humoral response directed towards this epitope [8–17]. The mechanisms of protection induced by M2e based vaccine differ from the CIV currently on the market, which is an egg-based split vaccine adjuvanted with alum [2]. The protection induced by the commercial CIV is based on neutralizing antibodies directed to the hypervariable regions located in the head of hemaglutinin (HA), as in the human split vaccine and provide sterilizing immunity [18]. It is anticipated that the effectiveness of CIV will decline over time as circulating virus shift and drift, as in humans [19]. However, a vaccine that targets the highly conserved influenza antigen M2e do not protect through sterilizing immunity and is rather based on antibody-dependent cell-mediated cytotoxicity (ADCC) [20]. In this model, immunoglobulin’s (preferentially IgG2a or IgG2c [21,22]) binds to the M2e peptide located at the surface of influenza infected cells [8], recruit and activate NK cells [21], and eradicate the influenza infected cell reservoirs [21]. Therefore, protection induced by the M2e-based vaccine depends on the effectors function of the IgG2a antibodies that eliminate virus-infected cells through activation of NK cells rather than through sterilizing immunity.

In this study, we propose the use of a novel vaccine platform made of the coat protein of a plant virus: malva mosaic virus (MaMV) [23,24]. MaMV is a new member of the potexvirus family and has a rod-shaped structure of approximately 540nm in length by 13nm in diameter [23]. Two other members of this plant virus family, namely potato virus X and papaya mosaic virus, have previously shown potential as vaccine platforms [8,25-33], adjuvants [8,34,35] or as modulators of innate immunity [36]. Recently, MaMV nanoparticles produced and purified from a recombinant bacterial system have been showed to stabilize CTL epitopes and to induce cross-presentation on the MHC class I located at the surface of human antigen presentation cells [24]. In the present study, we focused on the induction of a humoral response directed to the M2e peptide fused to the MaMV vaccine platform to generate a flu vaccine with a broad spectrum of protection in dogs.

The use of an adjuvant to boost the immune response directed to a particular epitope is often critical to maximizing the efficacy of a vaccine formulation. Efficient adjuvants that can trigger innate immunity are preferred over alum for the development of novel flu vaccines [18]. The outer membrane porins (OMPs) formulation purified from the bacterial membrane of *Salmonella typhi* containing both, the OmpF and OmpC porins has been reported to be TLR2-4 agonist, able to trigger innate immunity [19]. This formulation was showed to be a potent immunomodulator that can increase the immune response induced to *Micobacterium vaccae* [20], suggesting a good potential as an adjuvant.

This manuscript describes our efforts to combine the MaMV vaccine platform, the universal M2e antigen and the adjuvant OmpC to create a potent vaccine formulation able to induce a broad protection against influenza disease in dogs.

## Results

### Production of MaMV nanoparticles harboring the H3N8 M2e fusion

**Figure 1:**
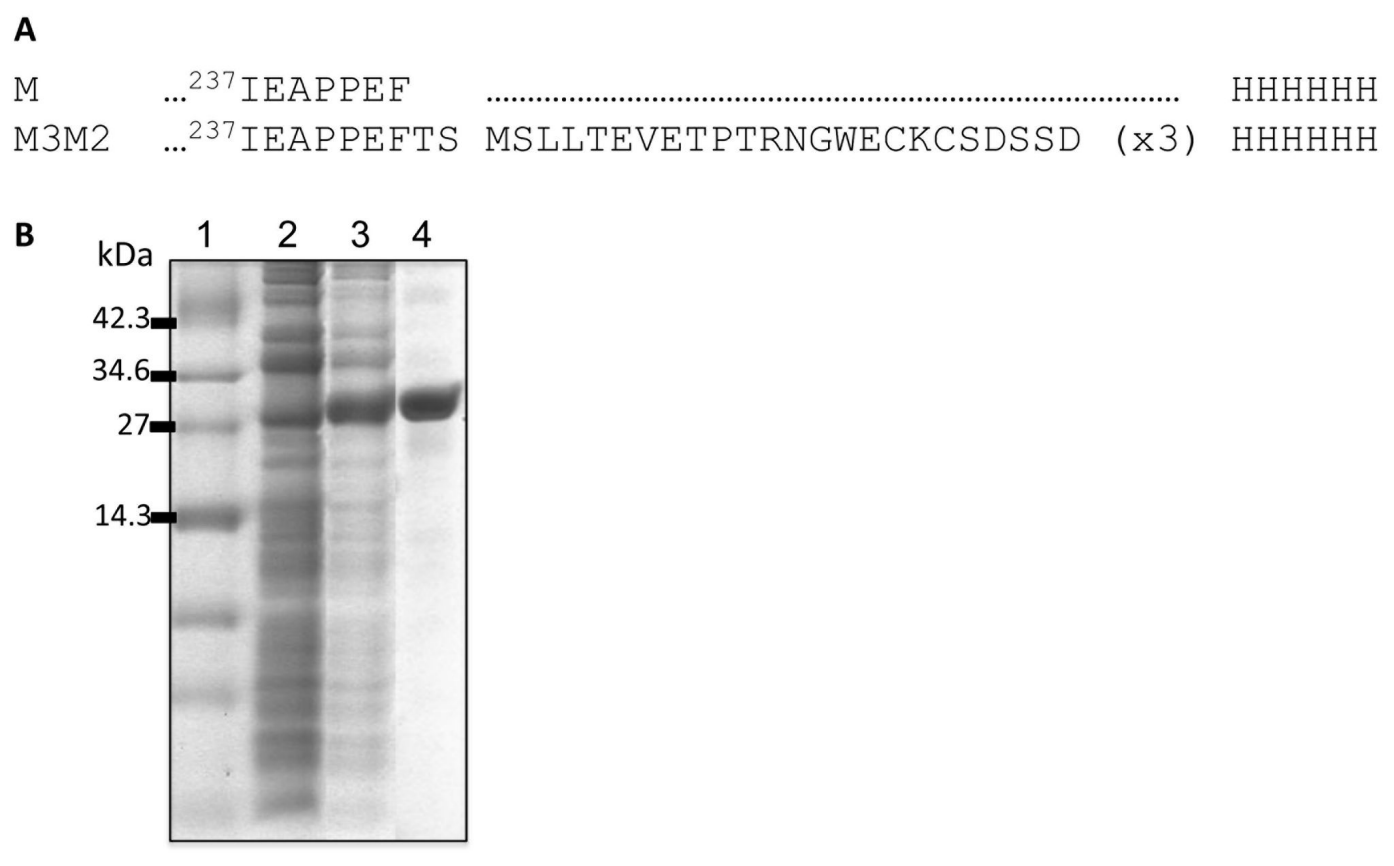
Expression and purification of M3M2 nanoparticles. (A) A fusion of a 3X tandem copy of the H3N8 M2e peptide was cloned at the C-terminus of the malva mosaic virus (MaMV) coat protein (CP), followed by a 6XH tag for ease of purification, to generate the recombinant fusion protein M3M2. (B) SDS-PAGE showing the expression and purification profile of the recombinant fusion protein expressed in *E. coli* (BL21DE3) from the expression vector pET3D. Upon addition of 1mM IPTG, expression of large amounts of the M3M2 protein was induced in the bacteria. Lanes: 1-molecular weight markers, 2-bacterial lysate without induction with IPTG, 3-bacterial protein lysate after 16 hours induction with IPTG, and 4-purified M3M2 used for immunization.

An alignment of the M2e peptides of influenza viruses isolated from infected dogs from the database (178 viral isolates) allowed us to find 172 isolates that showed an entirely conserved M2e peptide. Only 6 sequences diverged by one amino acid from the consensus (Figure S1). Therefore, the peptide M2e is a good choice for development of a vaccine with broad spectrum of protection to H3N8 influenza strain. We chose the consensus sequence of M2e (MSLLTEVETPTRNGWECKCSDSSD) and fused a 3xM2e tandem copy follow by a 6xH tag to the Malva mosaic virus (MaMV)-CP C-terminus (Figure 1A). We evaluated the capacity of MaMV-CP to tolerate the fusion and generate immunogenic nanoparticles. A stable fusion protein was produced and purified at high yield (100mg/L) and high purity (>95%) (Figure 1B). The purified recombinant protein was found as nanoparticles (M3M2) of 35nm in length as observed by dynamic light scattering (DLS) (Figure S2). DLS measures the length of the nanoparticles directly in solution; results were similar and reproducible for the 3 batches of M3M2 produced.

### The MaMV vaccine platform improves the immunogenicity of the H3N8 M2e peptide

**Figure 2:**
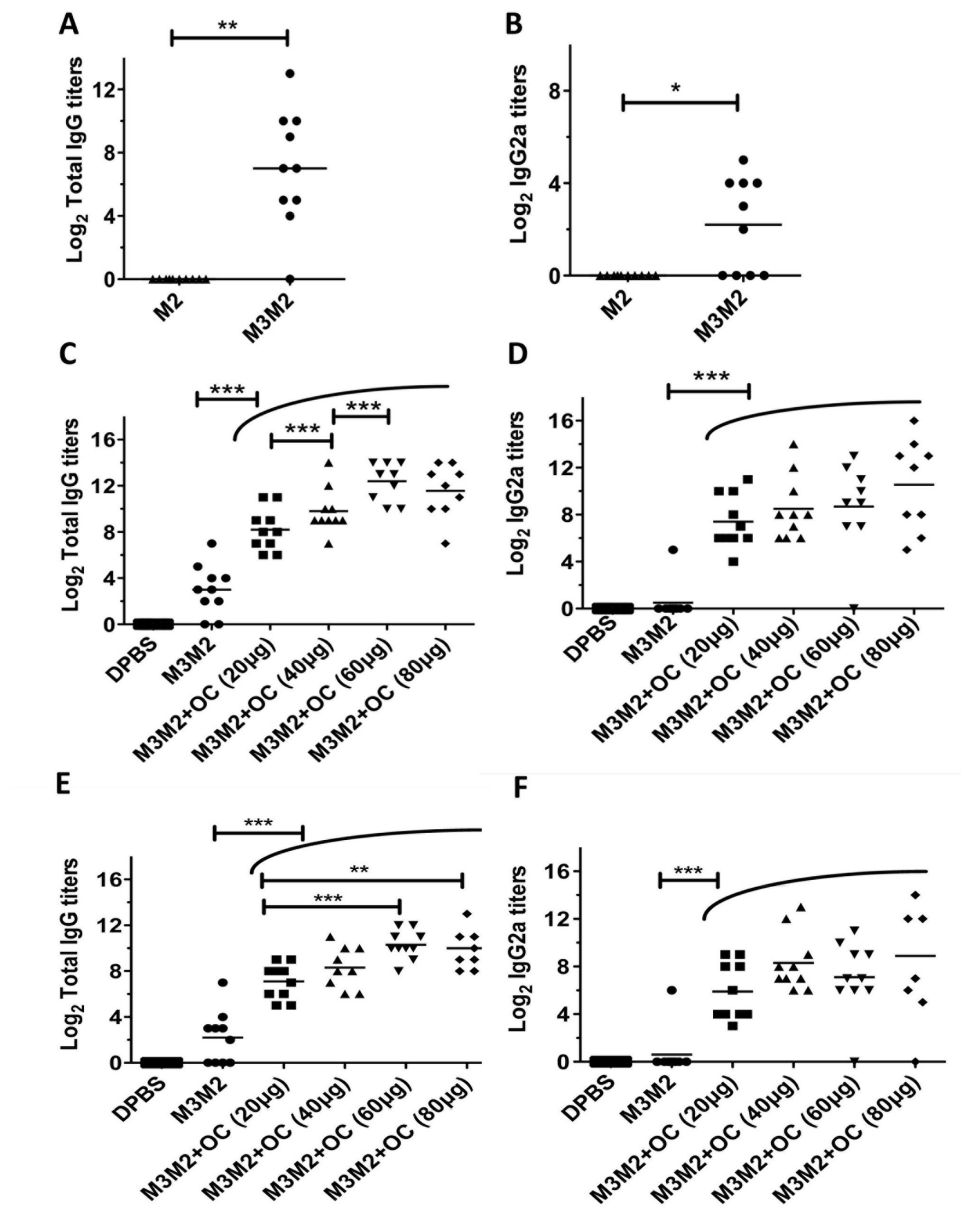
The M3M2 antigen mixed with the OmpC adjuvant induces a strong humoral response to the H3N8 M2e peptide. Balb/C mice (10 per group) were immunized twice with either 100µg of the recombinant M3M2 antigen or with 23µg of the H3N8 M2e peptide. Total IgG titers (A) or IgG2a (B) directed to the H3N8 M2e peptide were evaluated by ELISA using serum collected 14 days after the second immunization. Using a similar immunization protocol, Balb/C mice were immunized with 20µg of M3M2 alone or mixed with one of several doses of the adjuvant OmpC (OC) (20, 40, 60 or 80µg) to increase the immune response to the M2e peptide. Total IgG (C) and IgG2a (D) were evaluated from serum collected 14 days after the second immunization. Finally, to evaluate if the vaccine formulations could trigger a memory response, ELISA was performed with serum collected at day 61 after the second immunization of the same animals as in C and D. Total IgG (E) and IgG2a (F) directed to the peptide H3N8 M2e were measured. Adjuvanted groups unified under one arc means that they are all significantly different from the non-adjuvanted group with the same level of confidence showed with the above capped line. * p< 0.05, ** p< 0.01, *** p< 0.001.

To evaluate if fusion to the MaMV vaccine platform improved the immune response directed towards the M2e peptide, we immunized Balb/C mice (10 /group) twice at 14-day intervals by the subcutaneous (s.c.) route with 100µg of M3M2 nanoparticles or with 23µg of the M2e peptide alone (corresponding to the amount of M2e peptide found in fusion with the MaMV vaccine platform). An ELISA assay performed with serum harvested 14 days after the second injection showed that only mice immunized with the M3M2 constructs triggered a significant IgG response to the H3N8 M2e peptide (Figure 2A). An IgG2a response, that is wanted to trigger ADCC, was also detected in more than half of the animals vaccinated with M3M2 nanoparticles (Figure 2B). Immunization with the M2e peptide alone was not immunogenic (Figure 2AB).

### OmpC improved the immune response to the H3N8 M2e peptide

To increase the strength, quality and duration of the immune response directed towards the M2e peptide, we used purified OmpC porins (OC) as an adjuvant, tested at several doses. As before, Balb/C mice (10/group) were immunized s.c. twice at two-week intervals with M3M2 (20µg) alone, or together with increasing doses (20, 40, 60 or 80µg) of OC. The addition of the adjuvant in the vaccine formulation with M3M2 did not change the appearance of the solution, which remained clear for several hours. At all doses tested, the OC adjuvant improved the total IgG at day 17 after the second immunization (Figure 2C) significantly as compared to the non-adjuvanted M3M2 group. We also observed significant differences between the group adjuvanted with 20, 40 and 60µg of OC. The saturation of the total IgG response was reached with 60µg of OC since no significant differences were observed between the groups adjuvanted with 60 and 80µg (Figure 2C). We also found that all the adjuvanted groups showed significantly higher IgG2a titers than the M3M2 group. However, the adjuvanted groups were not significantly different between themselves (Figure 2D). Based on those results, the formulation containing 20µg M3M2 and 60µg OC was selected as our optimal formulation in mice. To evaluate if the vaccine formulations were able to trigger a memory response, an ELISA using serum harvested 61 days after the second immunization revealed that levels of total IgG remained significantly higher in the adjuvanted formulations as compared to the non-adjuvanted M3M2 group (Figure 2E). The adjuvanted groups adjuvanted with either 60 or 80µg of OC remained significantly higher than the group adjuvanted with 20µg of OC (Figure 2E). As for the ELISA at 17 days (Figure 2D), the IgG2a titers between adjuvanted groups were not significantly different but all of them were significantly higher than the non-adjuvanted M3M2 group (Figure 2G), suggesting that a memory response is triggered and could potentially provide protection to disease for at least 2 months.

### A vaccine formulation containing M3M2 and OC triggers a broad immune response

**Figure 3:**
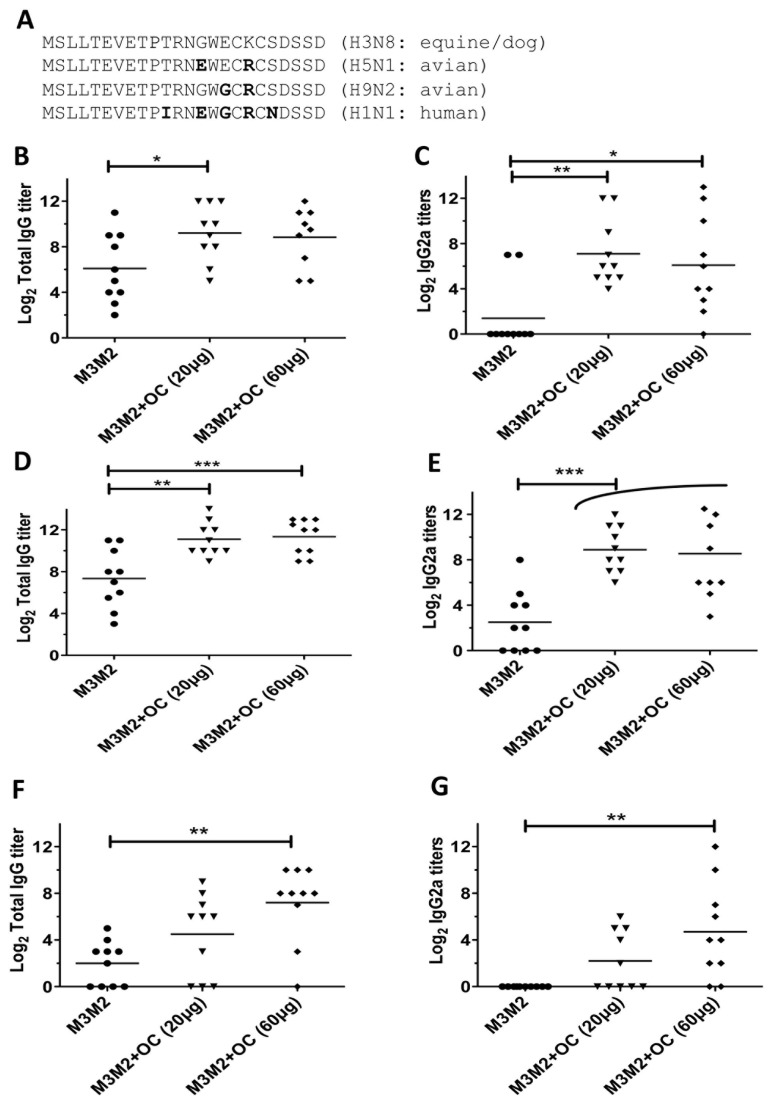
The serum obtained from mice immunized with the optimized M3M2/OC vaccine shows cross reactivity with divergent M2e peptides. (A) Amino acid sequence of the divergent M2e peptides derived from the avian H5N1, H9N2 or the human H1N1 strain. The serum used to perform ELISA was collected from mice immunized in the experiment shown in Figure 2A. Total IgG directed (B) IgG2a to H5N1 M2e, total IgG (C) and IgG2a (D) to H9N2 M2e and total IgG (E) and IgG2a (F) to the H1N1 M2e. * p< 0.05 and *** p< 0.001.

The M2e peptide of the strains A/Canine/Florida/43/2004 (H3N8) is highly conserved in all strains of influenza known to infect dogs, and diverges slightly from M2e of the avian H5N1, H9N2 and the human H1N1 (Figure 3A). The human M2e is the most divergent from the dog sequence, with four substitutions compared to the H3N8 sequence (Figure 3A). To evaluate if the vaccine formulations we used can generate antibodies able to recognize divergent M2e sequences, we performed an ELISA assay using serum harvested 17 days after the second immunization presented in Figure 2CD against the three divergent M2e peptides. The results revealed that the sera that have reached an average of log_2_ total IgG titer of 12 against the H3N8 peptide (Figure 2C) could reach a titer of 9 when ELISA was performed against the heterosubtypic H5N1 M2e peptide (Figure 3B). Sera with log_2_ IgG2a titers of 8 against the H3N8 M2e maintained an average titer of 7 when ELISA was made against the H5N1 peptide (Figure 3C). ELISA also revealed that a log_2_ total IgG titer of 12 (Figure 3D) and IgG2a titer of 8 (Figure 3E) could be reached against the H9N2 M2e. Those titers are as high as those obtained against the native H3N8 M2e (Figure 2CD). Finally, and as expected, ELISA against the most divergent human H1N1 M2e peptide gave the lower log_2_ total IgG titers with 8 (Figure 3F) and IgG2a titer with 4 (Figure 3G) as compared to 12 (total IgG) and 8 (IgG2a) respectively for the H3M8 M2e peptide (Figure 2CD). The adjuvanted formulation with 60µg OC gave the best results overall. In all cases, the adjuvanted groups gave significantly higher signals in the ELISA assay than the M3M2 non-adjuvanted group.

### The M3M2/OC vaccine induces a broad protection to influenza disease

**Figure 4:**
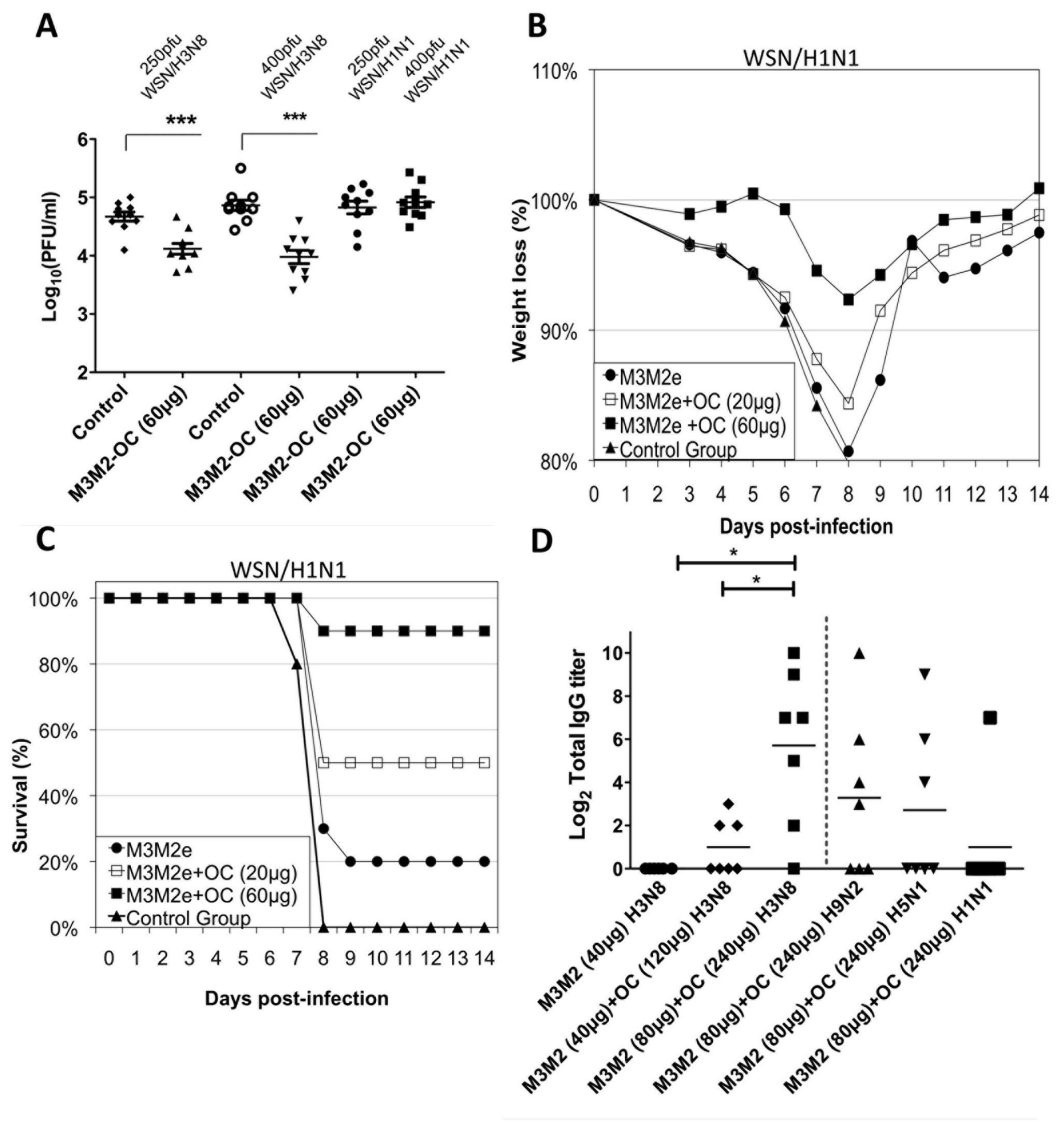
The optimized M3M2/OC vaccine induces a strong and broad protection to infection with homo- and hetero-subtypic influenza strains. Balb/C mice (10 per group) were immunized three times s.c. at 14-day intervals with saline (control) or with the optimized vaccine formulation [M3M2 (20µg)+OC (60µg)], and challenged 14 days following the third immunization with either 250 or 400 plaque forming units (pfu) of the homotypic WSN/H3N8 or the heterosubtypic strain WSN/H1N1. The lungs of the animals were collected on day 6 post-infection. Virus titration was performed in homogenized lungs in MDCK cells. Data are expressed as log_10_ of plaque forming units (pfu) (A). As before, Balb/C mice (10 per group) were immunized twice s.c. at 14-day intervals with either saline (control group), 20µg M3M2 alone or adjuvanted with either 20 or 60µg OC. At day 14 after the third immunization, mice were challenged with 100pfu of WSN/H1N1. Percentage weight loss (B), survival (C) and symptoms (Figure S2) were recorded for 14 days after the challenge. Twenty beagle dogs were immunized three times at 21-day intervals with 40µg M3M2 (6 dogs), 40µg M3M2+120µg OC (7 dogs) or 80µg M3M2+240µg OC (7 dogs) by the intramuscular route. Serum was collected 21 days after the third immunization and ELISA was performed against the M2e peptide H3N8. Also, ELISA against the M2e peptide of the strains H9N2, H5N1 H1N1 performed with the serum of dogs immunized with 80µg M3M2+240µg OC are showed at the right of the dotted lines (D). *p<0.05, ** p< 0.01 and ***<0.001.

Encouraged by the strong humoral response obtained with the vaccine formulation M3M2/OC, we next evaluated its capacity to induce protection to an influenza disease. Because the influenza H3N8 strains we tested are unable to infect mice efficiently, we chose to modify the mouse-adapted WSN/33 influenza strain, which is known to provide a fast and sustained infection in this animal model [37]. The M2e peptide of the WSN/33 strain is identical to the human peptide shown in Figure 3A that is 85% identical to influenza strains infecting humans [17,38] but is divergent from the H3N8 M2e (Figure 3A). Using the reverse genetic system of WSN/33, we modified the WSN/33 strain and introduced four amino acid substitutions into the M2e sequence to make it 100% identical to the dog H3N8 M2e (Figure 3A). The resulting mutated virus (WSN/H3N8) was at least as infectious as the WSN/33 (or WSN/H1N1) virus in mice (Figure S3). We used this modified virus to infect mice that were vaccinated three times s.c. at 14-day intervals with the optimal formulation [M3M2 (20µg)+OC (60µg)]. Infection with lethal doses was performed 14 days after the last injection using either 250 or 400pfu (plaque forming units) of the homologous WSN/H3N8 virus or the heterosubtypic WSN/H1N1 virus. At day 6 after infection, the animals were sacrificed and the lungs collected to evaluate virus titer. We found a significantly lower amount of the WSN/H3N8 virus in the lungs of animals vaccinated with the optimal vaccine as compared to the control group (Figure 4A). However, vaccinated mice were not protected from infection with the heterosubtypic strain WSN/H1N1 at either 250 or 400pfu.

The inoculums used in the previous challenge experiment were 100% lethal and induced a very strong infection. Since mice vaccinated with the optimized vaccine formulation induced production of antibodies able to bind the H1N1 M2e peptide (Figure 3FG), we hypothesized that this cross-reactivity is sufficient to provide protection to WSN/H1N1 while using a lower dose for the challenge. To test this hypothesis, mice (10/group) were vaccinated three times s.c. at 14-day intervals with either M3M2 (20µg) alone or adjuvanted with either 20 or 60µg of OC. Mice were infected with lower inoculums (100pfu) of the WSN/H1N1 strain, which still corresponds to a 100% lethal dose. Symptoms (Figure S4), percentage weight loss (Figure 4B), and survival (Figure 4C) were monitored for 14 days following the challenge. Mice vaccinated with the optimized formulation (20µg M3M2 + 60µg OC) exhibited 90% survival of the infection, very limited weight loss and only mild symptoms. The strength of the protection was linked to the amount of OC adjuvant added to the M3M2 antigen since the use of 20µg OC was not effective to induce protection (Figure 4B).

### The M3M2/OC vaccine is immunogenic in dogs

This vaccine was developed for the dog market, as dogs have recently become a new reservoir of influenza virus [1]. Therefore, we immunized dogs (6-month-old beagles) three times by the intramuscular route (i.m.) at 21-day intervals with either M3M2 (40µg) alone (6 dogs), M3M2 (40µg)/OC (120µg) (7 dogs) or M3M2 (80µg)/OC (240µg) (7 dogs). No adverse reaction was observed at the site of injection, either at the time of injection or 24 hours following treatment, for any of the treatments performed. Blood was collected 21 days after the third immunization and an ELISA assay against the H3M8 M2e peptide was performed. The results revealed that it is harder to mount a humoral response to the M2e peptide in dogs than in mice since the non-adjuvanted group failed to induce a detectable immune response (Figure 4D). The animals immunized with a dose of 80µg M3M2+240µg OC showed the most promising results since 6 dogs out of 7 triggered a significantly higher humoral response than when 40µg of M3M2 or 40µg M3M2+120µg OC were used. We also detected cross-reactive total IgG titers in animals immunized with the 80µg M3M2+240µg OC against the H9N2 M2e peptide (4 out of 7 animals), the H5N1 peptide (3 out of seven animals) and the H1N1 M2e peptide (1 out of seven animals) respectively. This result highlights the important role of the adjuvant in the vaccine formulation in dogs.

## Discussion

We have shown that the MaMV vaccine platform is able to stabilize the H3N8 M2e peptide and improve its immunogenicity. In addition, we showed that OmpC could be used as an adjuvant. OmpC increased and expanded the antibody response to the M2e peptide, leading to production of cross-reactive immunoglobulin’s (total IgG and IgG2a) in mice and in dogs (tot IgG) that are able to bind to M2e peptides of the divergent strains (H9N2, H5N1 and H1N1). The M2e peptides harbor two major immunogenic regions: the first is central to the peptide and corresponds to the peptide EVETPIRNE [39]. A better cross-reactivity was observed to the H9N2 M2e peptide because it is identical to H9N2 M2e. The H5N1 M2e was also cross-reactive since it shows only one amino acid substitution at the C-terminus of the central peptide (G^14^E). Cross-reactivity to H1N1 M2e is weaker, probably because the central peptide harbors two substitutions (T^11^I and G^14^E). Nevertheless, a weaker but positive cross-reactivity was measured by ELISA against H1N1 M2e peptide that provided protection in a mouse model. This is probably related to antibodies that are directed a second immunogenic region of the M2e peptide that is entirely conserved throughout the M2e of all influenza A strains, e.g., the SLLTEVET region located at the N-terminus. A monoclonal antibody capable of inducing protection to a challenge with a H1N1 or a H5N1 influenza virus was found to bind this region [40], which supports our hypothesis. Infection of vaccinated mice with lethal doses of homotypic (WSN/H3N8) and heterosubtypic (WSN/H1N1) strains of influenza virus in a mouse model indicated that cross-reactive antibodies can also provide protection to influenza disease. The vaccine formulation was also showed to be immunogenic in dogs showing a lower, but measurable level of cross reactivity to the M2e peptides with the H9N2 and the H5N1 peptides. It is still uncertain if those levels are sufficient to provide protection against disease in dog.

It is well recognized that M2e based vaccines trigger eradication of influenza infected cells through ADCC (20). Our data are consistent with ADCC since we show a moderate reduction (about 10 fold) of virus titers in vaccinated mice rather than a sterilizing immunity as those induced by neutralizing antibodies with the CIV usually through immunoglobulin’s directed to variable regions of the hemaglutinnin (HA). Therefore, with this type of vaccine, influenza infection must be initiated before the immunity acquired by the vaccine start to show its benefice. The major advantage with our M2e vaccine on the CIV is in the broad protection that is induced with our formulation since this M2e peptide is highly conserved through almost all (172 out of 178; Figure S1) the H3N8 strains known to infect dogs to date.

Our vaccine formulation is produced entirely in bacteria. We therefore anticipate that the vaccine can be produced in bulk at reasonable cost and has a commercial potential. Alternatively, this formulation could be combined with the current CIV to produce a vaccine that induces both mechanisms of protection, i.e., sterile immunity (IgG directed to HA) and ADCC directed to M2e. Additional benefits can be expected from a CIV+OC+M3M2 formulation because the adjuvant OmpC could potentially increase the immune response directed to HA (dose sparing) as previously reported using another adjuvant [35].

## Materials and Methods

### Cloning and production of M3M2 nanoparticles and OmpC

The bacterial expression plasmid pET3D expressing the MaMV coat protein (CP) gene in *E. coli* BL21DE3 has been described previously [17]. A unique *Spe*I site located at the C-terminus of the MaMV CP allowed cloning of a tandem trimer of the H3N8 M2e coding sequence in fusion with MaMV CP (Figure 1A). The production and purification procedure for the fusion protein (M2M2) was identical to that described previously [23,24]. The adjuvant OmpC was purified from the *Salmonella typhi* strains STYF302 (OmpF^–^) [41] following an established protocol [42].

### SDS-PAGE and dynamic light scattering (DLS)

Samples were mixed with one-third of the final volume of loading buffer containing 5% SDS, 30% glycerol and 0.01% bromophenol blue. SDS-PAGE was performed as described elsewhere [24]. The size of nanoparticles was recorded with a ZetaSizer Nano ZS (Malvern, Worcestershire, United Kingdom) at 20°C at a concentration of 0.1 mg/ml diluted in PBS 1X. The variation in nanoparticle size induced by temperature variation was measured under the same experimental conditions. The graph in Figure 1C shows measurements of three separate experiments performed on three different batches.

### Immunization and infectious challenge

All immunizations were performed via the subcutaneous route (s.c.) in 6- to 8-week-old Balb/C mice. One or two booster shots were given at 14-day intervals and blood samples were always collected 14 to 17 days after each immunization. The infections were performed with the WSN/33 (H1N1) mouse-adapted influenza strain [37] or with the modified virus WSN/H3N8 harboring the M2e sequence of the H3N8 strain A/Canine/Florida/43/2004. Briefly, mice were infected by the intranasal route with 50 ml sample containing different concentration of virus. Mice were monitored daily for clinical symptoms of infection (loss of body weight, abnormal behavior and ruffled fur). Death was recorded over a period of 14 days.

### ELISA quantification

ELISA was performed as described elsewhere [8]. H3N8 M2e (MSLLTEVETPTRNGWECKCSDSSD), H9N2 M2e (MSLLTEVETPTRNGWGCRCSDSSD), H5N1 M2e (MSLLTEVETPTRNEWECRCSDSSD) and H1N1 M2e (MSLLTEVETP**I**RNEWGCRCNDSSD) peptides were synthesized by GLBiochem Shanghai Ltd, Shanghai, China, and resuspended in endotoxin-free PBS (Sigma, St. Louis, MO). Costar High Binding 96-well plates (eBioscience, San Diego, CA) were coated overnight at 4°C with 100µl/well of the M2e peptide diluted to a concentration of 1µg/ml in 0.1M NaHCO_3_ buffer (pH 9.6). Results are expressed as an antibody endpoint titer, defined as when the OD value is 3-fold greater than the background value obtained with a 1:50 dilution of serum from PBS-injected mice. For Figure 2 C to F, Figure 3 B to G and Figure 4 A and D, the statistical analysis of the groups were performed with a parametric ANOVA test followed by Tukey’s post-test to compare differences among groups of mice. For Figure 2A that contained only 2 groups, the statistical analysis was made with the Wilcoxon matched pairs test. Values of *p<0.05, **p < 0.01 and ***p < 0.0001 were considered statistically significant. Statistical analyses were performed with GraphPad PRISM 5.01.

### Ethics statement

All work with animals was performed using an ethics protocol approved by the 'Comité de Protection des Animaux - CHUQ (CPA-CHUQ)' of our Institution. The approval for this project can be found under the authorization number 2010148-1.

## Supporting Information

Figure S1 **Alignment of the M2e peptide from 178 different canine influenza viruses (CIV) sequenced to now.** The sequence of the M2e peptide is identical in 172 out of 178 sequences available on the Influenza Virus Ressource of the NIH (www.ncbi.nlm.nih.gov/genomes/FLU/Dataset). The sequence of the 6 virus that differed only by one amino acid with the conserved sequence are showed. The amino acid substitution with the common sequence of the A/Canine/Florida/43/2004 are showed in bold and underlined.
(TIF)

Figure S2 **Measure of the size of the M3M2 nanoparticles.** The graph show 3 readings made on three different batches using the dynamic light scattering (DLS).
(TIF)

Figure S3 **Infectivity of WSN/H1N1 and WSN/H3N8 virus in a Balb/C mouse model.** Balb/C mice (10 per group) were infected with either 100, 250 and 400 pfu of the WSN/H3N8 or WSN/H1N1 virus. Survival with the WSN/H3N8 virus (A) or the WSN/H1N1 virus (C), and percentage weight loss with the WSN/H3N8 virus (B) or the WSN/H1N1 virus (D) was monitored for 9 days after intranasal challenge.
(TIF)

Figure S4 **Symptoms developed during the challenge of immunized mice.** Development of symptoms was scored everyday after the challenge. Symptoms/ 0 : No symptoms, 1 : Lightly spiked fur, slightly curved back, 2: Spiked fur, curved back, 3: Spiked fur, curved back, difficulty in moving and mild dehydration, 4 : Spiked fur, curved back, difficulty in in moving, severe dehydration, closed eyes and ocular secretion.
(TIF)
