# Supplementary figures and images for: A Novel M2e Based Flu Vaccine Formulation for Dogs
Source: PLoS One. 2013 Oct 2;8(10):e77084. doi: 10.1371/journal.pone.0077084 (PMC3788766; doi:10.1371/journal.pone.0077084)

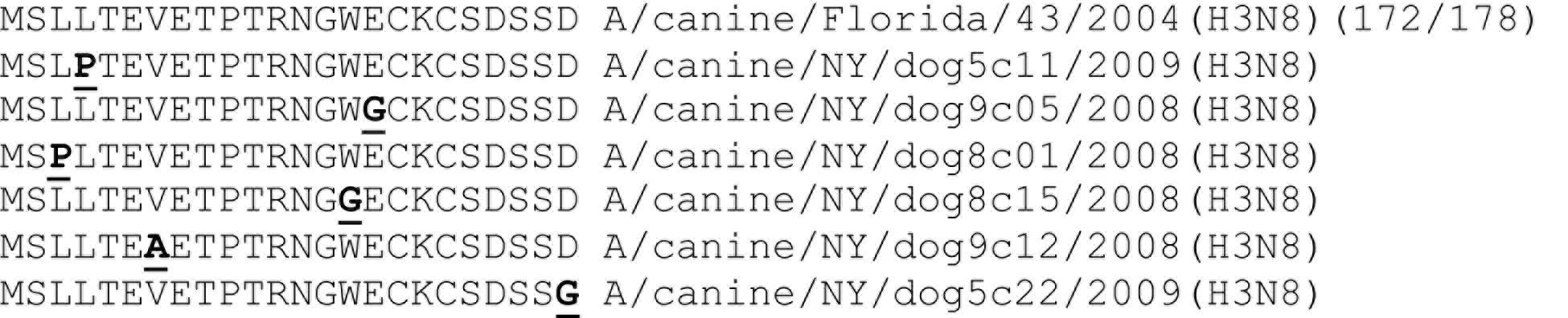

Supplement: Figure S1 — Alignment of the M2e peptide from 178 different canine influenza viruses (CIV) sequenced to now. The sequence of the M2e peptide is identical in 172 out of 178 sequences available on the Influenza Virus Ressource of the NIH (www.ncbi.nlm.nih.gov/genomes/FLU/Dataset). The sequence of the 6 virus that differed only by one amino acid with the conserved sequence are showed. The amino acid substitution with the common sequence of the A/Canine/Florida/43/2004 are showed in bold and underlined. (TIF) [file pone.0077084.s001.tif]

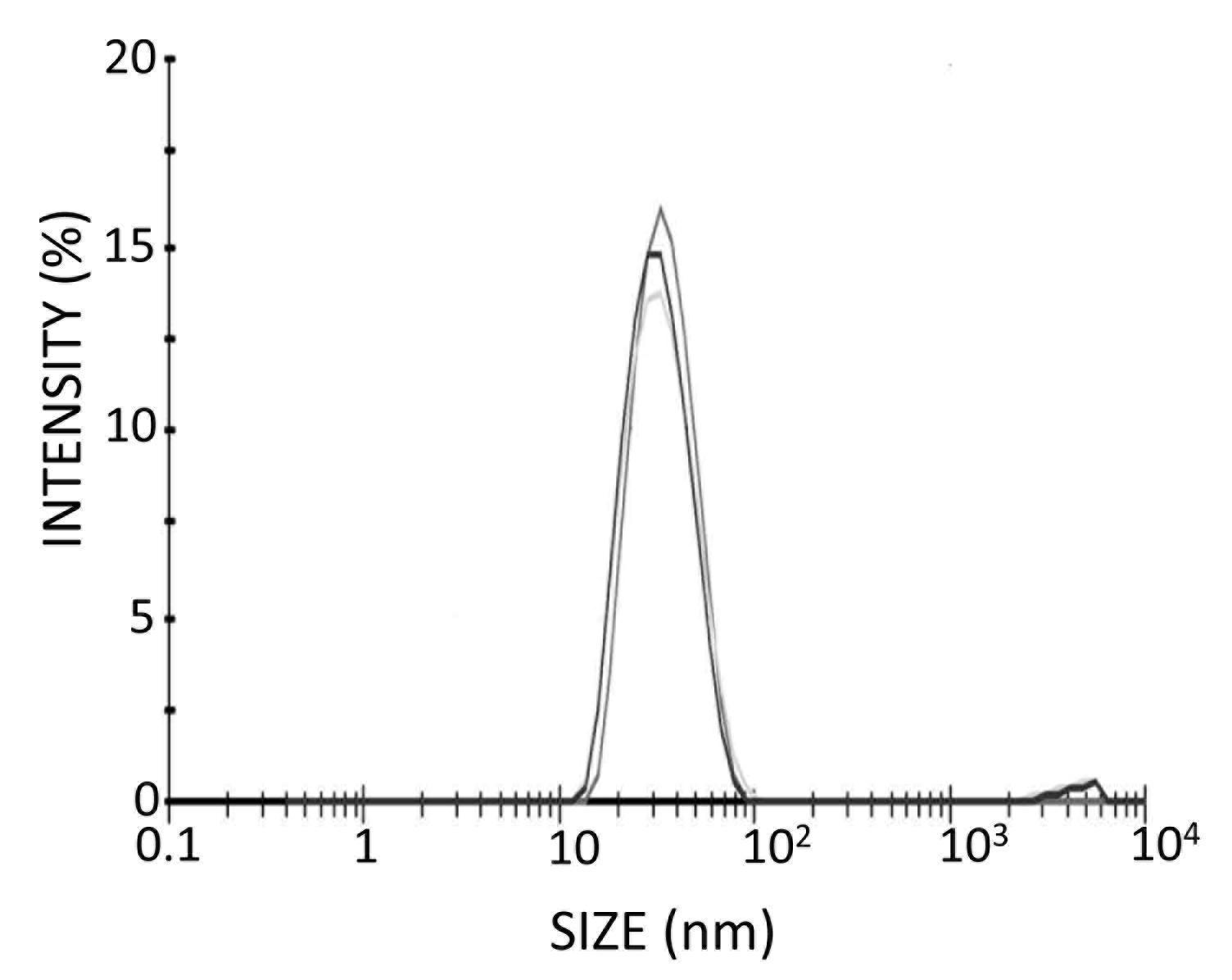

Supplement: Figure S2 — Measure of the size of the M3M2 nanoparticles. The graph show 3 readings made on three different batches using the dynamic light scattering (DLS). (TIF) [file pone.0077084.s002.tif]

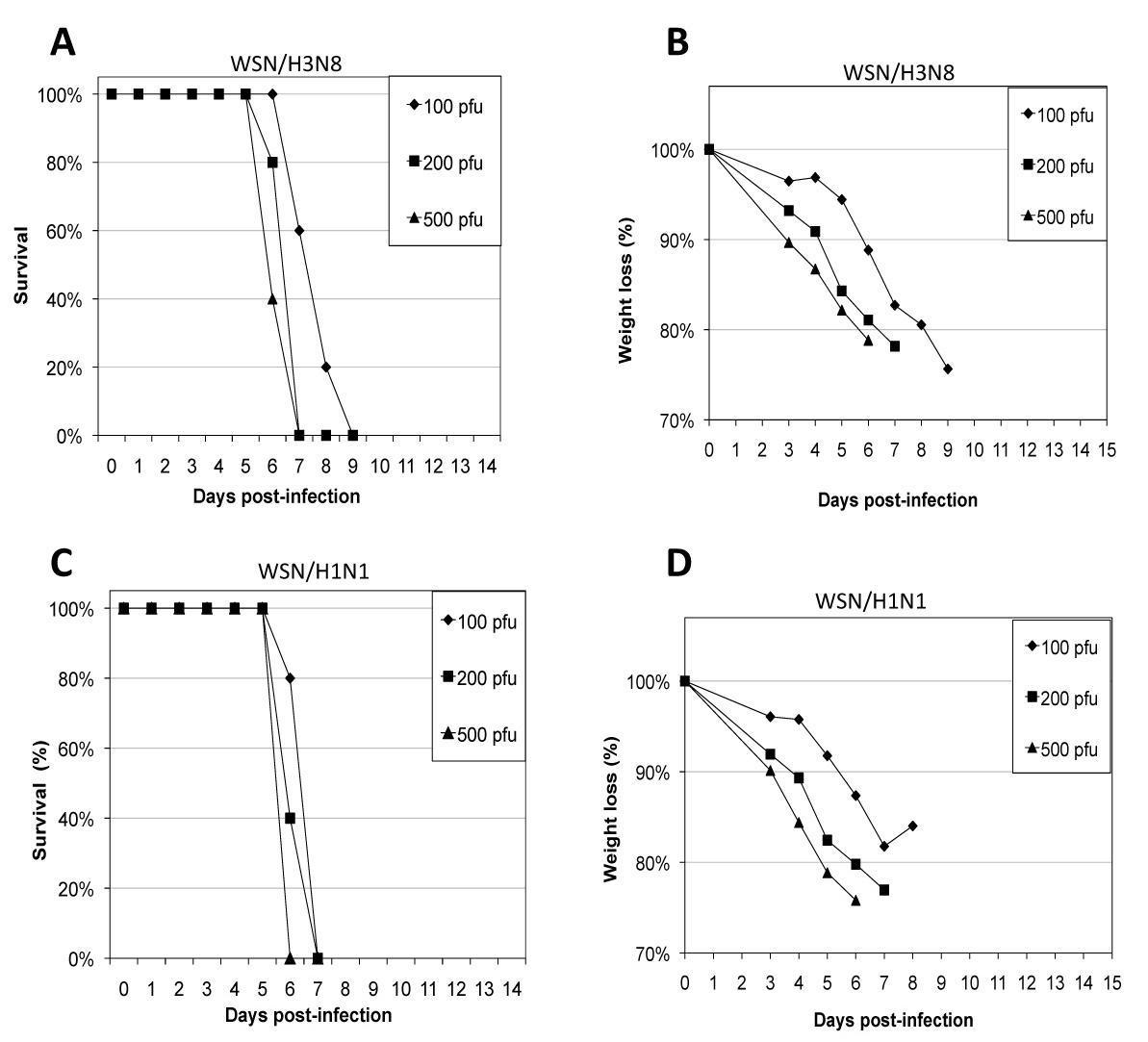

Supplement: Figure S3 — Infectivity of WSN/H1N1 and WSN/H3N8 virus in a Balb/C mouse model. Balb/C mice (10 per group) were infected with either 100, 250 and 400 pfu of the WSN/H3N8 or WSN/H1N1 virus. Survival with the WSN/H3N8 virus (A) or the WSN/H1N1 virus (C), and percentage weight loss with the WSN/H3N8 virus (B) or the WSN/H1N1 virus (D) was monitored for 9 days after intranasal challenge. (TIF) [file pone.0077084.s003.tif]

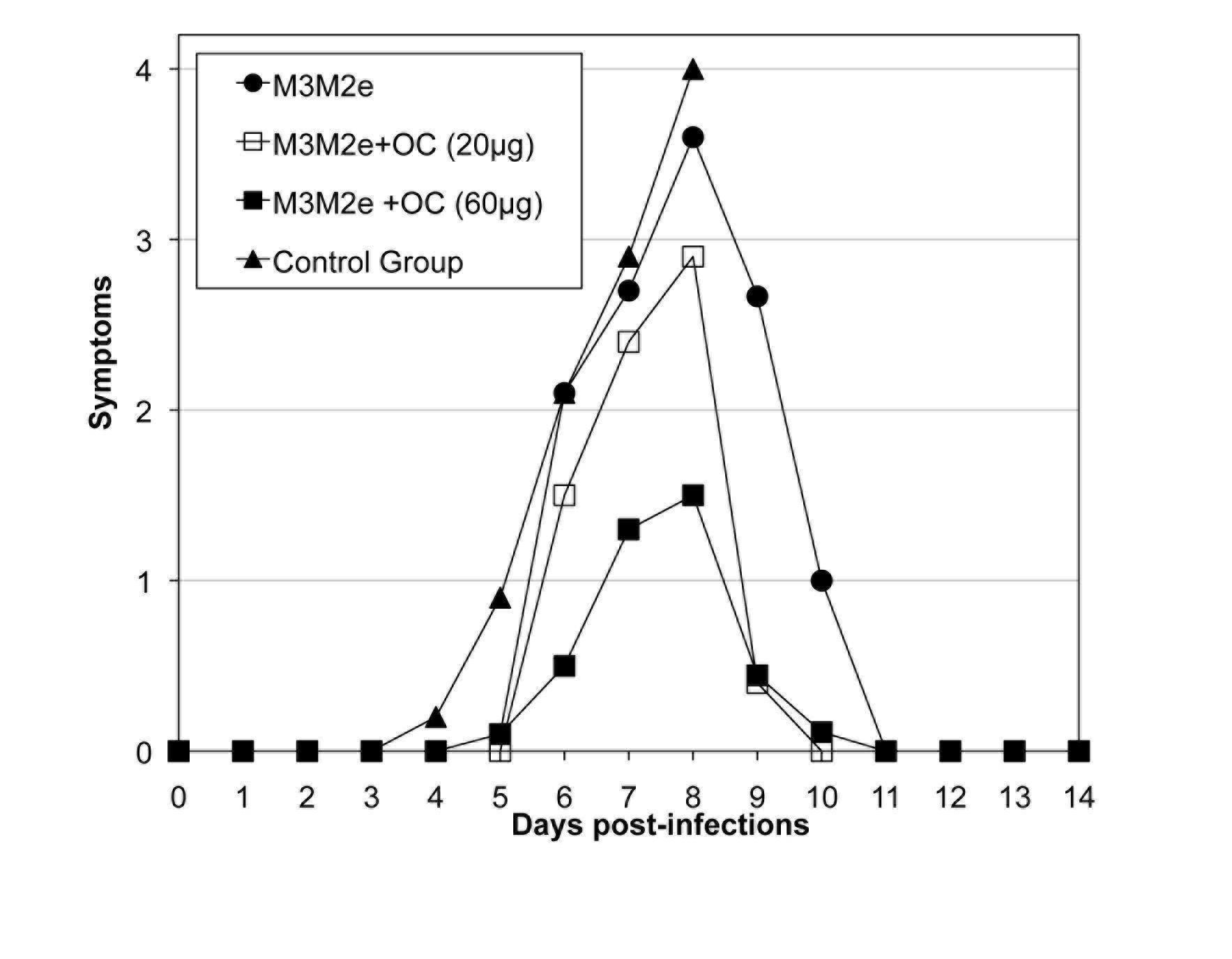

Supplement: Figure S4 — Symptoms developed during the challenge of immunized mice. Development of symptoms was scored everyday after the challenge. Symptoms/ 0 : No symptoms, 1 : Lightly spiked fur, slightly curved back, 2: Spiked fur, curved back, 3: Spiked fur, curved back, difficulty in moving and mild dehydration, 4 : Spiked fur, curved back, difficulty in in moving, severe dehydration, closed eyes and ocular secretion. (TIF) [file pone.0077084.s004.tif]
